# Transcriptome analysis of epithelioma papulosum cyprini cells after SVCV infection

**DOI:** 10.1186/1471-2164-15-935

**Published:** 2014-10-25

**Authors:** Junfa Yuan, Yi Yang, Huihui Nie, Lijuan Li, Wangang Gu, Li Lin, Min Zou, Xueqin Liu, Min Wang, Zemao Gu

**Affiliations:** Department of Aquatic Animal Medicine, College of Fisheries, Huazhong Agricultural University, Wuhan, 430070 People’s Republic of China; Freshwater Aquaculture Collaborative Innovation Center of Hubei Province, Wuhan, 430070 People’s Republic of China; Key Lab of Freshwater Animal Breeding, Ministry of Agriculture, Wuhan, 430070 People’s Republic of China; Department of Immunology, Zunyi Medical University, Zunyi, 563003 People’s Republic of China

## Abstract

**Background:**

Spring viraemia of carp virus (SVCV) has been identified as the causative agent of spring viraemia of carp (SVC) and it has caused significant losses in the cultured common carp (*Cyprinus carpio*) industry. The molecular mechanisms that underlie the pathogenesis of the disease remain poorly understood. In this study, deep RNA sequencing was used to analyse the transcriptome and gene expression profile of EPC cells at progressive times after SVCV infection. This study addressed the complexity of virus–cell interactions and added knowledge that may help to understand SVCV.

**Results:**

A total of 33,849,764 clean data from 36,000,000 sequence reads, with a mean read length 100 bp, were obtained. These raw data were assembled into 88,772 contigs. Of these contigs, 19,642 and 25,966 had significant hits to the NR and Uniprot databases where they matched 17,642 and 13,351 unique protein accessions, respectively. At 24 h post SVCV infection (1.0 MOI), a total of 623 genes were differentially expressed in EPC cells compared to non-infected cells, including 288 up-regulated genes and 335 down-regulated genes. These regulated genes were primarily involved in pathways of apoptosis, oxidative stress and the interferon system, all of which may be involved in viral pathogenesis. In addition, 8 differentially expressed genes (DEGs) were validated by quantitative PCR.

**Conclusions:**

Our findings demonstrate previously unrecognised changes in gene transcription that are associated with SVCV infection *in vitro*, and many potential cascades identified in the study clearly warrant further experimental investigation. Our data provide new clues to the mechanism of viral susceptibility in EPC cells.

**Electronic supplementary material:**

The online version of this article (doi:10.1186/1471-2164-15-935) contains supplementary material, which is available to authorized users.

## Background

Spring viraemia of carp virus (SVCV), the causative agent of spring viraemia of carp (SVC), is classified as a member of the family Rhabdoviridae and belongs to the genus *Vesiculovirus*. SVC is an important disease affecting cyprinids, primarily common carp (*Cyprinus carpio*). This high-mortality disease is endemic in Europe, North America, and parts of Asia [1]. The major clinical signs for SVCV infection include ascites, and petechiae and ecchymoses in the gills and skin [2].

SVCV exhibits the typical bullet-shaped morphology of a vertebrate rhabdovirus. Its genome is a linear single-stranded, negative-sense RNA that is approximately 11 kb in length and encodes five structural proteins: nucleoprotein (N), phosphoprotein (P), matrix protein (M), glycoprotein (G), and RNA-dependent polymerase (L) [3, 4].

There have been several studies concerning the pathogenic mechanism (s) involved in SVCV infection have been reported [5–8]. Our previous work demonstrated that the expression of heme oxygenase 1 (*HO-1*) was down-regulated during SVCV infection *in vivo*, which suggested that SVCV infection could induce host oxidative stress that might contribute to tissue injury [7]. High-throughput methods, including pathway-targeted microarrays and proteomic analysis, have also been used to scan the host response to viral infection [9, 10]. These studies suggest that apoptosis, oxidative stress and the interferon (IFN) system may contribute to the mechanisms of the viral pathogenesis. However, a comprehensive identification of the genes involved in viral pathogenesis as well as major signal transduction pathways and intracellular interaction networks remains unavailable. Ultrahigh-throughput sequencing technologies permit genome-wide transcriptome analysis at high resolution, and these technologies have been widely used to study pathogenic processes during virus infection, including infections by aquatic viruses [11, 12].

New evidence has suggested that the current lineages of the epithelioma papulosum cyprinid (EPC) cell line originated from the fathead minnow (*Pimephales promelas*) [13]. The temperature growth range, good splitting ratio (1/10) and virus susceptibility make EPC cells highly suitable both for fish pathology and comparative virology studies. The fathead minnow, a species of temperate freshwater fish belonging to the cyprinid family, is widely used as an indicator of environmental water monitoring in ecotoxicological research [14, 15]. However, only sporadic genetic information about EPC cells or the fathead minnow is available, which might limit further studies with these resources.

In the present study, we present the results from sequencing and assembly of the transcriptome of EPC cells at progressive times after SVCV infection. Genes involved in oxidative stress, apoptosis, and the IFN system as well as nearly all major conserved metazoan signal transduction pathways were largely identified in the EPC transcriptome. It is the first light upon this well known and used fish cell line in fish virology. Additionally, a great number of genes that were differentially expressed upon SVCV infection were obtained and functionally annotated. The gene expression patterns for some of these genes were verified by RT-qPCR. These results offer insight into the complexity of the virus–cell interactions, and add new information that may help to control SVCV infection.

## Results

### Transcriptome sequence assembly and analysis

**Figure 1:**
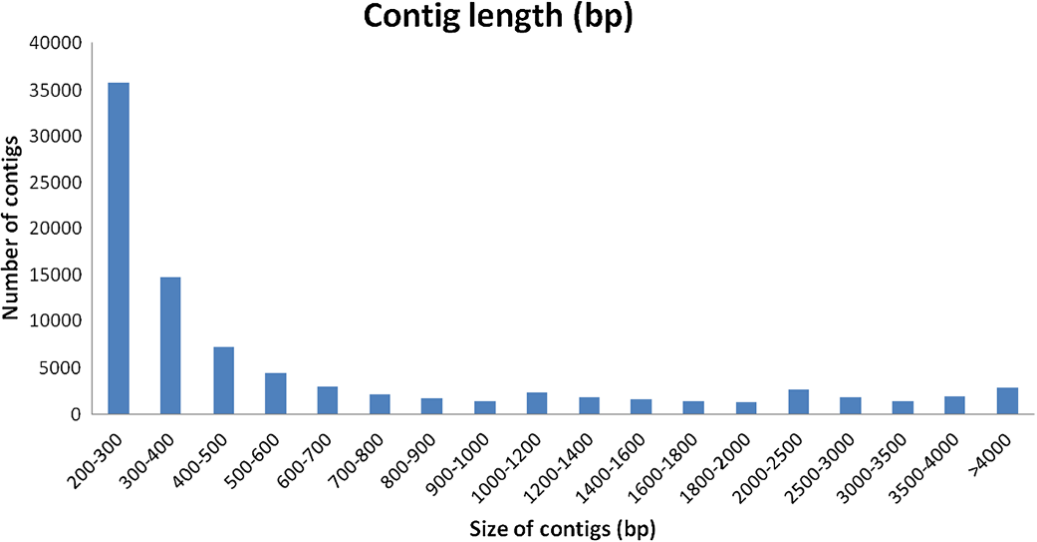
**Assembled contig length distribution of the** ***Pimephales promelas*** **transcriptome.** The x-axis indicates contig size and the y-axis indicates the number of contigs of each size.

To obtain an overview of the transcriptome of the EPC cells, cDNA samples from normal EPC cells and from EPC cells at different stages during SVCV infection were mixed and sequenced on an Illumina machine. A total of 33,849,764 clean data from 36,000,000 sequence reads, with a mean read length 100 bp, was obtained. These raw data were assembled into 88,772 contigs. The mean contig size was 831 bp, with lengths ranging from 201 bp to 17,900 bp. The contig size distribution is shown in Figure 1.

**Figure 2:**
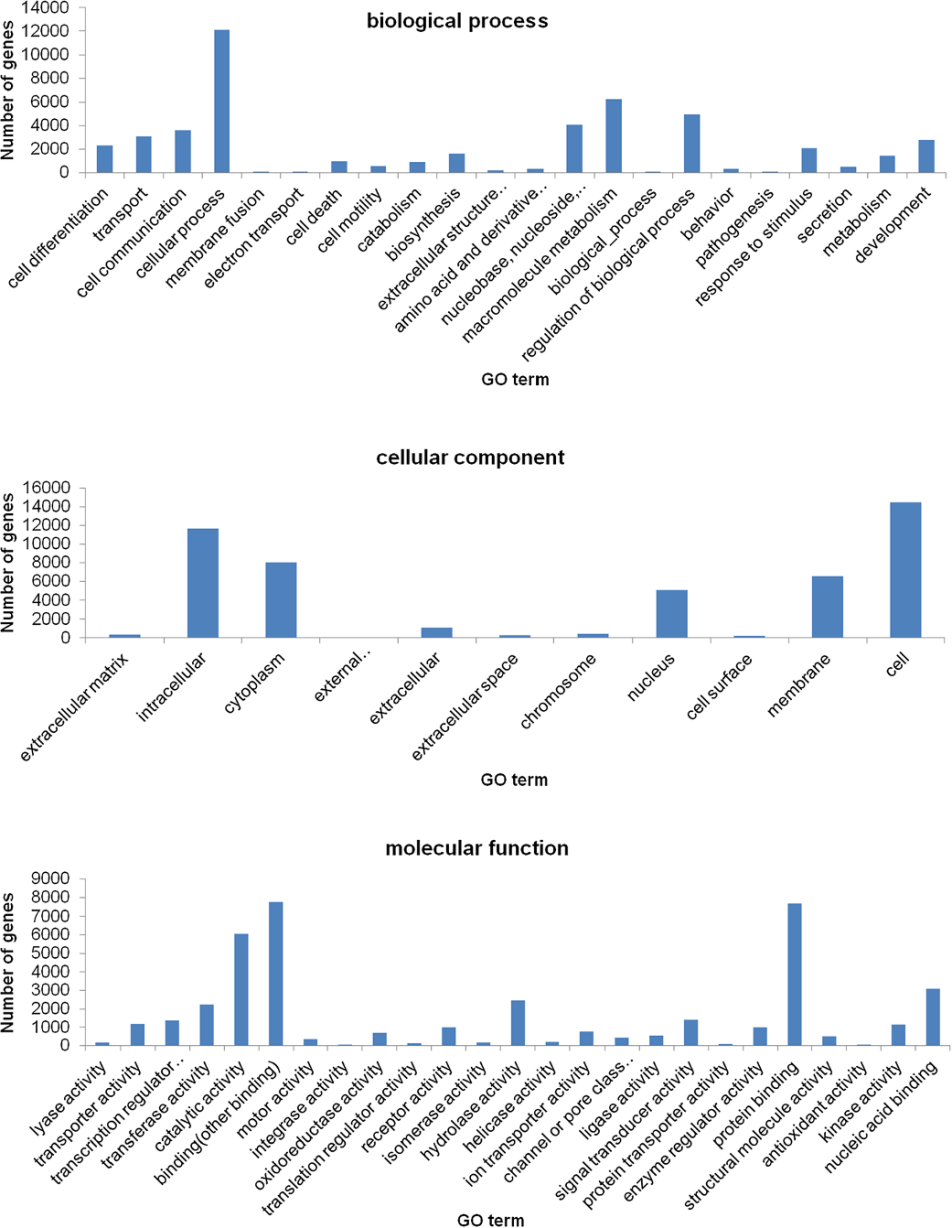
**Gene ontology assignments for** ***P. promelas.*** The annotated contigs from *P. promelas* sequencing that matched the three major categories, including biological process, cellular component, and molecular function were shown. The x-axis indicates the GO terms and the y-axis indicates the number of genes mapped to the indicated GO term.

**Figure 3:**
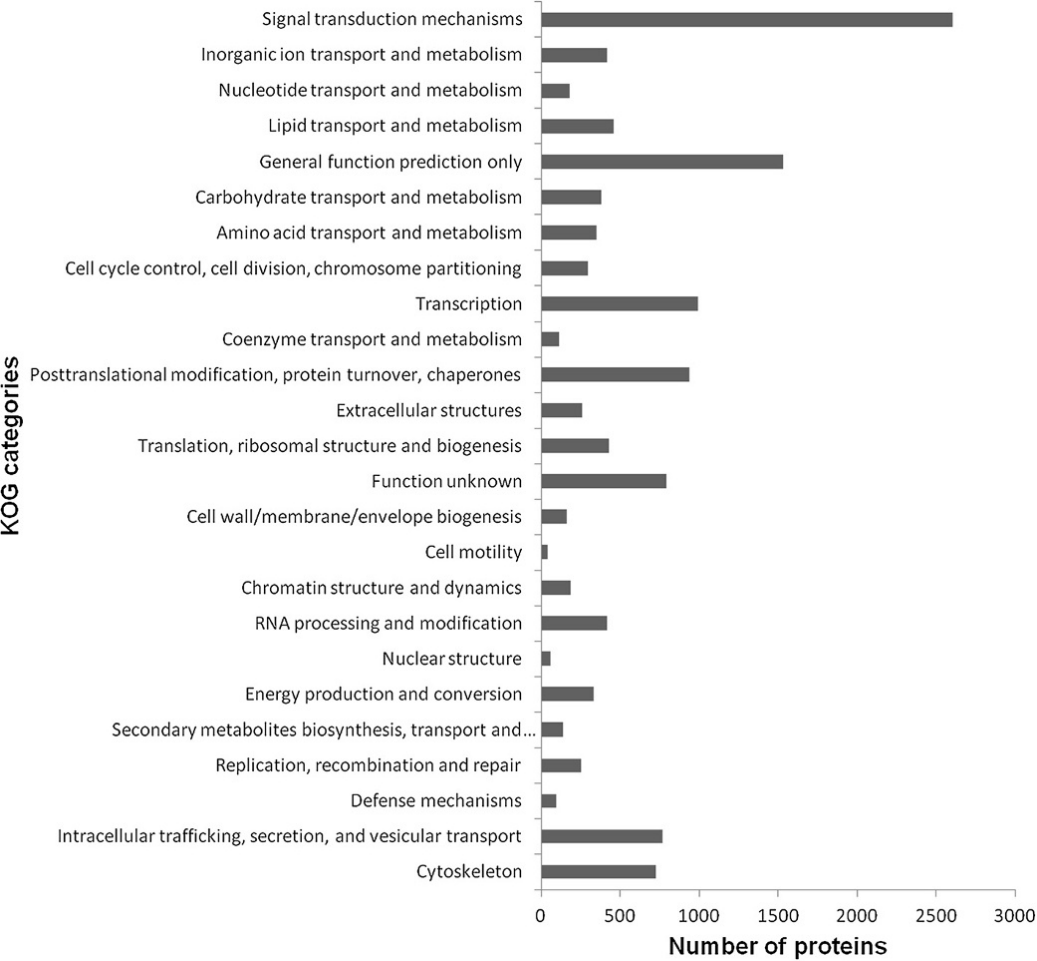
**KOG classification of the** ***P. promelas*** **transcriptome.** A total of 12,896 predicted proteins have a KOG classification among the 25 categories. The x-axis indicates the number of predicted proteins and y-axis indicates the KOG categories.

**Figure 4:**
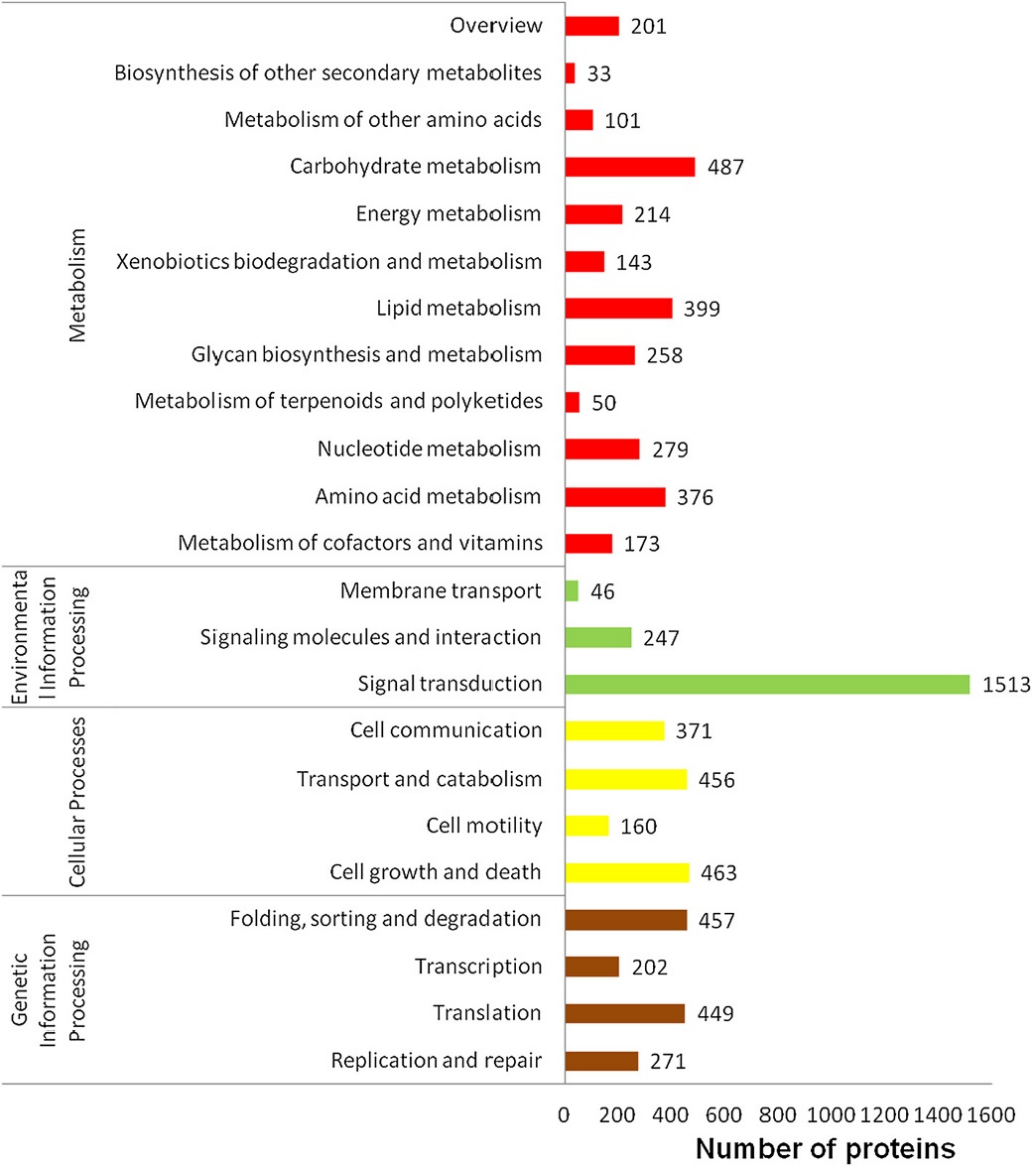
**KEGG classification of the** ***P. promelas*** **transcriptome.** The x-axis indicates the number of predicted proteins and the y-axis indicates the pathway.

BLASTX searches for all contigs from EPC cells were performed against several protein databases, including the GenBank non-redundant (NR) and Uniprot databases with an E- value cut-off of 10^−5^. Of the 88,772 contigs, 19,642 and 25,966 had significant hits to the NR and Uniprot databases respectively, and they respectively matched 17,642 and 13,351 unique protein accessions.Further gene ontology (GO) analysis was performed with these contigs. A total of 16,994 unique proteins mapped to 114,154 GO terms: 48,270 unigenes mapped to biological processes, 40,247 unigenes mapped to molecular functions, and 48,151 unigenes mapped to cellular components (Figure 2).EuKaryotic Orthologous Groups (KOG) classification of the unigenes is important for functional annotation and evolutionary studies. As shown in Figure 3, a total of 12,896 unigenes were finally mapped onto 25 different KOG categories. The largest KOG group was “Signal transduction mechanisms” (2,604 unigenes), followed by “General function prediction only” (1,531 unigenes) and “Posttranslational modification, protein turnover, chaperones” (937 unigenes).To obtain more information for predicted functions of the unigenes, the genes from the EPC cells were categorised in the Kyoto Encyclopedia of Genes and Genomes (KEGG) database. A total of 7,349 unigenes was obtained the KO number (Figure 4).

**Figure 5:**
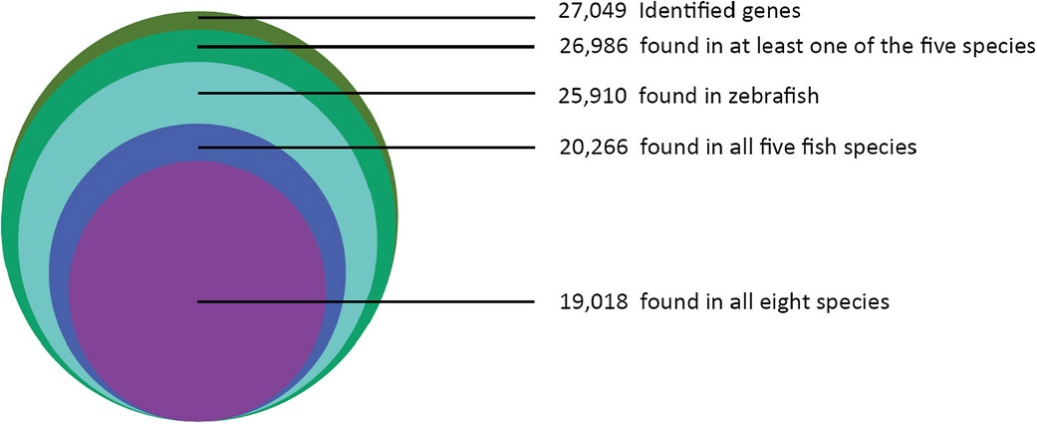
**Conservation of** ***P. promelas*** **gene identities with other species.** Number of *P. promelas* homologous genes identified from other species using BLASTX searches with an E- value cut-off of 10^−5^.

To assess the evolutionary conservation of the identified unique genes in EPC cells, the number of hits to unique genes in each species of zebrafish, medaka, Tetraodon, Fugu, stickleback, human, mouse, and chicken were compared. A total of 19,018 (70.3% of total number of unique fathead minnow genes) putative known unique genes was found in all eight species; 20,266 (74.9%) were found in all five fish species; and 26,986 (99.8%) were found in at least one of the five fish species (Table 1 and Figure 5), indicating a high level of conservation of gene content among *Pimephales promelas* and other teleost fish species.

**Table 1:** **Summary of the BLASTX (BLSATP) search analysis of** ***P. promelas*** **unique sequences**

Database	Hits*	Unique protein	% of total unique proteins
NR^1^	25596	17642	
Uniprot^1^	19642	13351	
**Refseq/Ensembl**			
Zebrafish	25910	15612	36.49% of 42787
Medaka	23006	12508	50.69% of 24674
Tetraodon	22059	12196	52.76% of 23118
Fugu	22539	14352	30.00% 0f 47841
Stickleback	22919	12934	46.90% of 27576
Human	21802	13534	12.97% of 104310
Mouse	21782	12422	24.15% of 51437
Chicken	21103	10178	62.24% of 16354
Cumulative unique^2^	27049	17396	

The version of indicated species of protein database is the ensemble release-73(ftp://ftp.ensembl.org/pub/release-73/fasta/).

*Number of significant alignments using all *P. promelas* unique sequences as queries to search the listed databases. ^1^Number of significant alignments using all *P. promelas* unique sequences as queries to search EMBOSS with BLASTp. ^2^Cumulative unique totals were derived from the sum of unique gene/protein identities across all listed species.

### Differentially expressed genes

To identify differentially expressed genes (DEGs) that potentially are involved in SVCV infection, cDNA libraries were constructed from pooled mRNAs extracted from the SVCV- infected EPC cells (3 h, 6 h and 24 h post infection) and non-infected groups. Subsequently, these libraries were sequenced on a Genome Analyzer II. DEGs were obtained based on the RPKM of the genes either in SVCV-infection or non-infection group. A gene with an RPKM ratio larger than 2 or smaller than 0.5 was considered to be a DEG [11, 12]. For the SVCV transcriptome, all five genes encoded by the SVCV genome, including N, P, M, G and L, were transcribed in EPC cells during the entire infection course, though the M and L gene were decreased at 6 h and 24 h post infection. The statistics of DEGs of host cells between different groups is shown in Table 2. Altogether, 623 genes were differentially expressed in EPC cells at 24 h post SVCV infection (1.0 MOI) compared to the non-infected cells, including 288 up-regulated genes and 335 down-regulated genes (Additional file 1: Table S2). Among the up-regulated genes, the expression levels of 177 genes were increased more than 4 times, and 58 genes were increased more than 16 times. *C-fos* and kruppel-like factor 2a (*KLF2A*), both associated with stress response, were the genes that showed the highest up-regulation, with an increase of more than 32 times. Among the down-regulated genes, the expression levels of 100 genes were decreased more than 4 times. The most down-regulated gene adrenomedullin 2 (*ADM2*), which associated with oxidative stress, was decreased more than 32 times.

**Table 2:** **Statistics of the differentially expressed genes (DEGs) upon SVCV infection between various time points**

Sample	Up-regulated	Down-regulated
3h Vs 0h	162	27
6h Vs 3h	11	75
6h Vs 0h	71	48
24h Vs 6h	276	360
24h Vs 3h	240	461
24h Vs 0h	288	335

### Functional annotation of the DEGs

**Figure 6:**
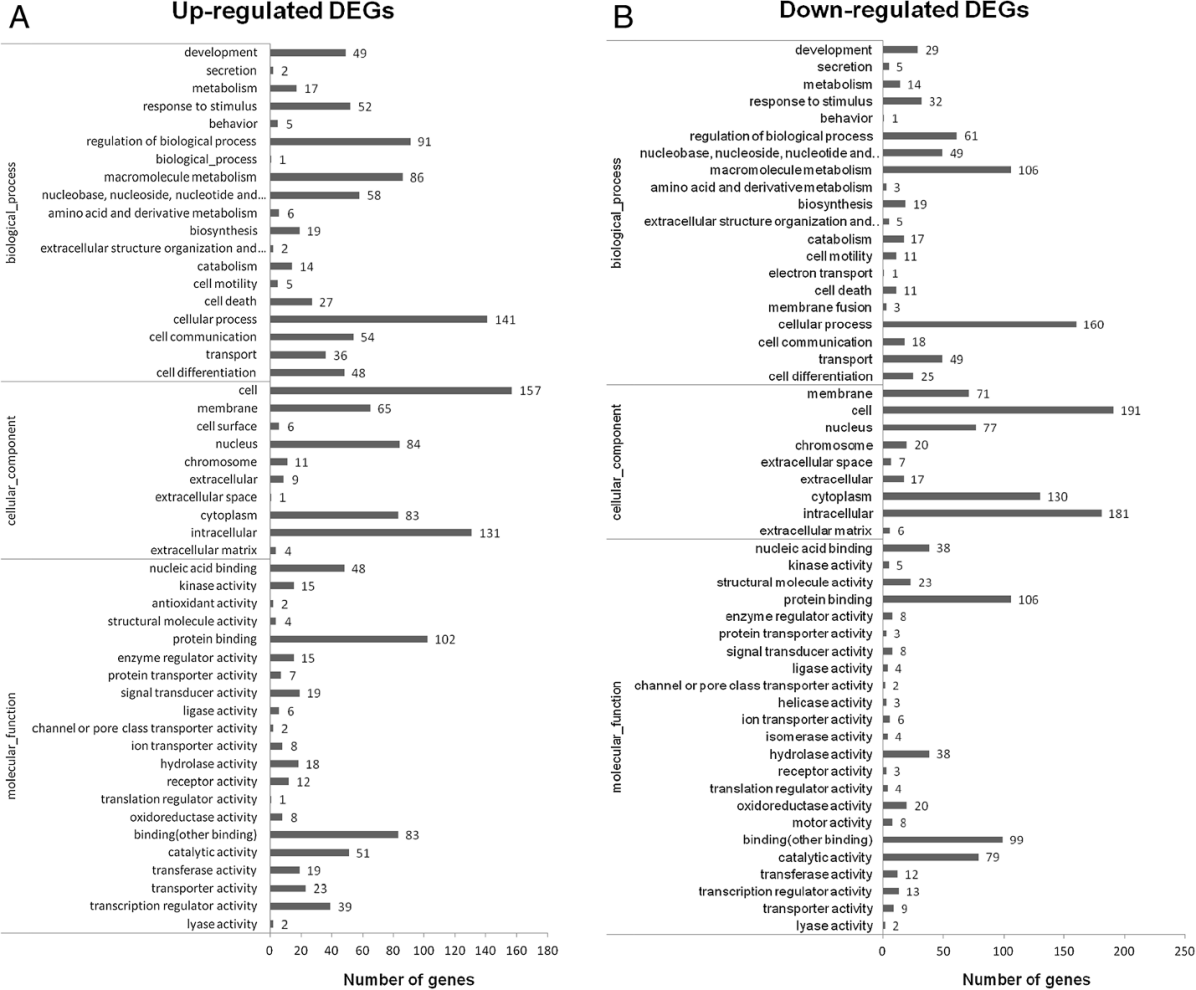
**Gene ontology assignments for differentially expressed genes (DEGs) upon SVCV infection.** The DEGs upon SVCV infection that matched various ontology (GO) categories, including biological process, cellular component, and molecular function. The x-axis indicates the GO terms and the y-axis indicates the number of genes. **A**, GO analysis for the up-regulated genes upon SVCV infection. **B**, GO analysis for the down-regulated genes upon SVCV infection.

**Figure 7:**
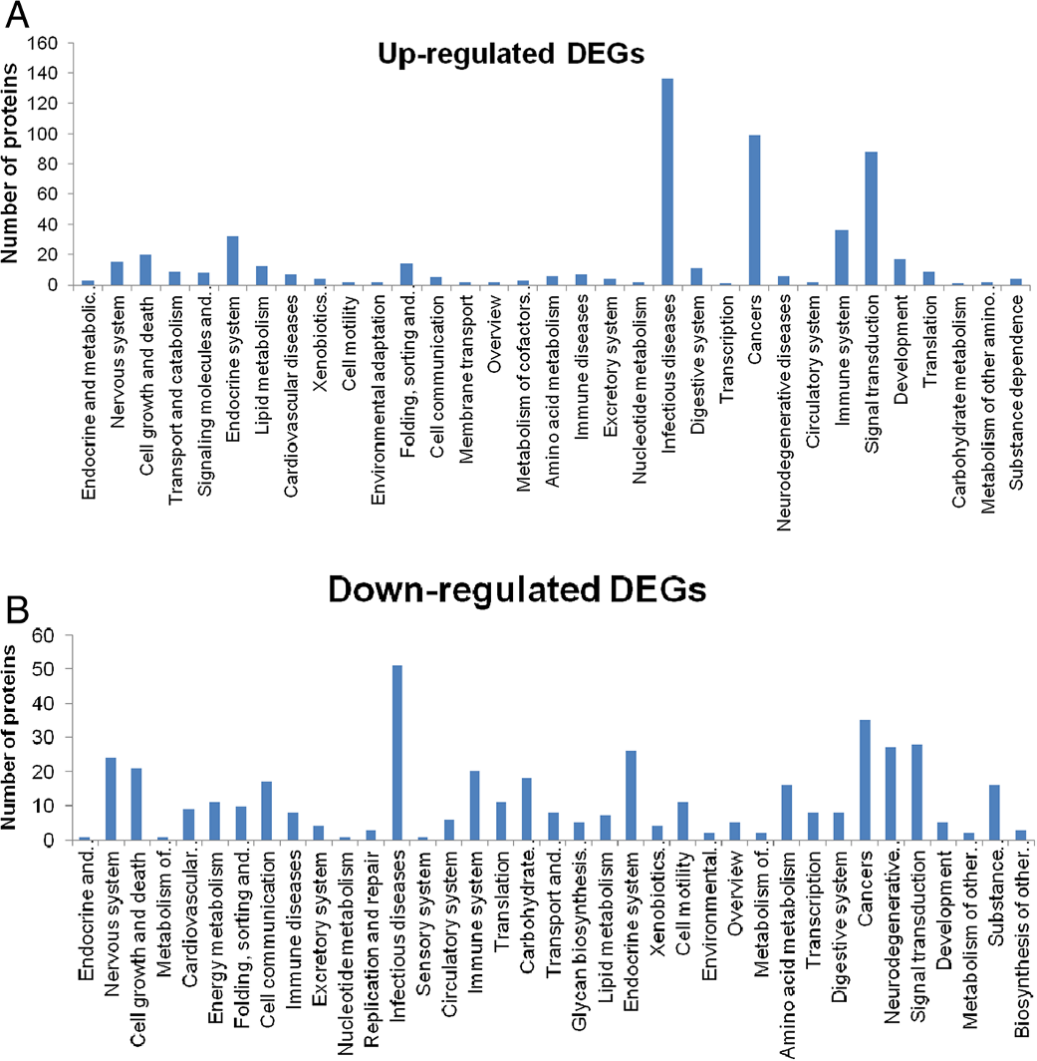
**KEGG classification of the differentially expressed genes (DEGs) upon SVCV infection.** The KEGG classification of up-regulated genes **(A)** and down-regulated genes **(B)** upon SVCV infection is shown. The x-axis indicates the pathway and the y-axis indicates the number of DEGs.

To understand the functions of the DEGs and the biological processes involved in SVCV infection, all of the DEGs were mapped to terms in the GO (Figure 6) and KEGG databases (Figure 7). GO analysis showed that a total of 1,748 unigenes had GO annotations. Among the up-regulated genes of host cells at 24 h post infection, 713 unigenes were mapped to biological processes, 551 unigenes were mapped to cellular components, and 484 unigenes were mapped to molecular functions. Of the biological process related genes, most were involved in cellular processes (141), regulation of biological processes(91), macromolecule metabolism (86), cell communication (58) and responses to stimulus (51). Most of the cellular component related genes were involved in the cell and nucleus. Most of the molecular function related genes were involved in binding (protein or other), transcription regulator and transporter activity. The GO analysis of the down-regulated genes at 24 h post infection is show in Figure 5B. Among these DEGs at 24 h post SVCV infection, “nucleus”, “intracellular” and “chromosome” in the cellular component ontology, “regulation of biological process”, “response to stimulus” and “macromolecule metabolism” in the biological process ontology, and “protein transporter activity” and “structural molecule activity” in the molecular function ontology were enriched by p-value analysis.KEGG analysis on the DEGs revealed that the up-regulated genes at 24 h post SVCV infection were assigned to 33 KEGG pathways (Figure 6). Nearly 50% percent of the up-regulated DEGs were assigned to the top 5 pathways as follows: infectious diseases (136), cancer (99), signal transduction (88), immune system(36) and endocrine system(32) (Q value < 0.05). Additionally, the down-regulated genes were assigned to 37 KEGG pathways, including infectious diseases (50), cancer (35), signal transduction (28), neurodegenerative diseases (27), and endocrine system (26). Altogether, the pathways involved with the DEGs that were enriched for the terms “cell growth and death”, “immune disease”, “infectious diseases”, “endocrine system”, “cancer”, “neurodegenerative diseases” and “signal transduction” were enriched by p value analysis.

### Kinetics of DEGs at different time points post- SVCV infection

Compared to the non-infected EPC cells, there were 162 up-regulated genes and 27 down-regulated genes at 3 h post infection (Table 2). When comparing DEGs of host cells between the groups of 6 h and 3 h post- infection, the number of up-regulated and down-regulated genes is 11 and 75, respectively. Looking at various genes, many were up-regulated at 3 h or 6 h, then decreased to the control level. This suggested that SVCV infection stirred up an obvious stress reaction in the early stage of infection. For instance, Interleukin-11b, involved in antibacterial and antiviral responses [16], was transiently and sharply up-regulated at 3 h by SVCV infection, and then decreased to the normal level at 6 h and 24 h. In contrast to the early stage of SVCV infection, DEGs with modest overlaps were found and resulted in cell damage.

### Verification of transcriptome data by RT-qPCR

**Figure 8:**
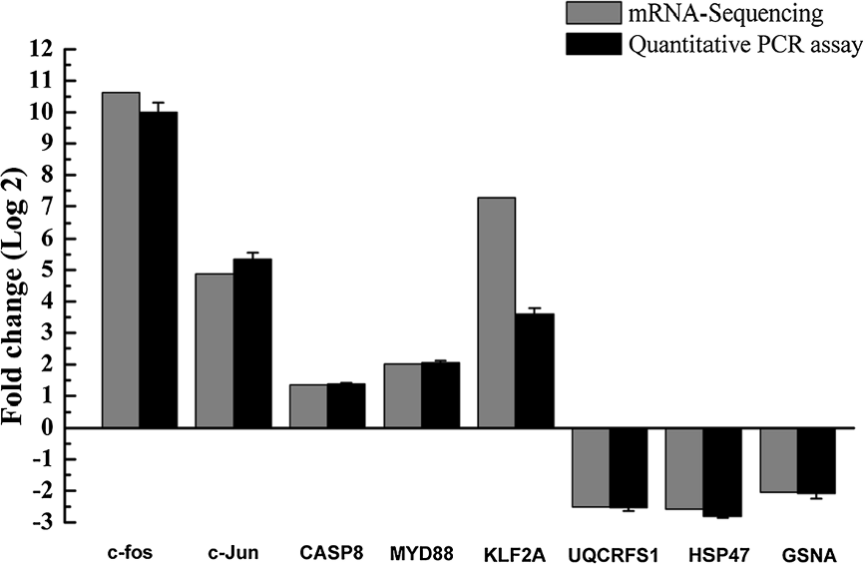
**RT-qPCR confirmation of the differentially expressed genes (DEGs) upon SVCV infection.** Relative transcript levels (fold changes) of selected DEGs were determined by the real-time PCR, using TATA box binding protein (*TBP*) as the reference control, and shown by the black bars. Data shown are the mean of triplicates ± SD. Three parallel experiments were performed and one representative experiment of three is shown. The transcript abundance from DEG data is shown by the grey bars. The minus value means the gene is down-regulated after SVCV infection; while the positive value means the gene is up-regulated in SVCV-infected cells.

To further evaluate our DEG library, 5 up-regulated DEGs and 3 down-regulated DEGs with different fold changes were randomly selected to perform RT-qPCR. Among these selected DEGs, *c-fos*, *c-Jun*, and *KLF2A* showed the highest up-regulation in mRNA sequencing method. *c-Jun* in combination with *c-fos*, forms the activator protein 1 (AP-1) early response transcription factor and is associated with stress response. The two other up-regulated genes were caspase 8 (*CASP8)* and myeloid differentiation primary response 88 (*MYD88*). These two genes were involved in the regulation of cell death and the innate immune response, respectively. The three selected down-regulated DEGs were heat shock protein 47 (*HSP47*), ubiquinol-cytochrome c reductase, Rieske iron-sulfur polypeptide 1(*UQCRFS1*) and GSNA. The RT-qPCR results revealed the same expression tendency as the DEG data, despite some quantitative differences in expression level (Figure 8). Among these selected DEGs, the obvious quantitative differences between sequencing and qPCR methods was only observed on the expression of *Klf2a*. Taken together, qRT-PCR analysis confirmed the tendency detected by the mRNA sequencing analysis.

## Discussion

### Global analysis of viral susceptibility genes in EPC transcriptome

The EPC cell line was established in the 1970s and has become one of the most widely used tools for research on fish virus and the diagnosis of fish diseases. Most of viruses causing systemic infections of fish families and amphibians, as well as Indiana-type vesicular stomatitis virus can propagate in EPC cells [13]. Mining the viral susceptibility genes from the transcriptome data may shed light on the potential mechanism for the broad spectrum sensitivity of this cell line to viruses. Previously, twelve independent studies were summarised to compile a list of human genes important for human immunodeficiency virus (HIV) and other virus infection [17]. A total of 388 human genes that were identified in two or more of these independent studies was suggested to be important for viral infection in mammalian cells. Among these, only 54 homologous genes lacked detectable expression levels in the EPC transcriptome (Additional file 2: Table S3). Of these unexpressed genes, several were specially expressed on mammalian T cells, natural killer (NK) cells or antigen presenting cells (APC), including CD2, CD4, CD4, CXCR4, CD44, CD86. For instance, CD2 is expressed on the surface of T cells and NK cells, and CD58 is expressed on APC, particularly macrophages. These two adhesion molecules are vitally important for hepatitis B virus (HBV) infection. The gene expression profile of EPC cells suggests that EPC would be one of the best cell models for the study of virus-host interactions. This contrasts with CHO-K1, a preferred host cell line for the production of therapeutic proteins, which is naturally resistant to several viral infection; in CHO-K1 there were 158 genes lacking detectable expression levels and 4 genes that were not found in its genome [18]. Interestingly, overlaps were found among the non- expressing genes in the EPC and CHO-K1 cell lines. For example, interleukin 1 alpha (IL1α), responsible for the production of inflammation, as well as the promotion of fever and sepsis, was not expressed in the EPC and CHO-K1 cells. Another gene of interest that was not expressed in EPC cells. is catalase, a common enzyme found in nearly all living organisms exposed to oxygen. Such information could aid an in-depth analysis of viral susceptibility genes in EPC cells.

### Overlaps among different screens for the host responses to SVCV infection

Until now, two independent studies have investigated the host response to SVCV infection by systematic methods [9, 10]. Liu *et al.* revealed 55 dynamically changed proteins in SVCV-infected EPC using 2-DE profile and MS identification [10]. Compared with our results presented here, a total of 10 genes (18%) overlapped with similar expression profile (Additional file 2: Table S4). Similarly, the overlap between our results and a study which was screened using pathway-target microarrays for zebrafish infected with and survived SVCV was modest [9]. In that study, 16 multipath genes common to more than 6 pathways were identified in 2-day exposed or 30-day survivors of SVCV infection. Among these 16 regulated genes, 7 genes shared a similar expression pattern. However, variation due to 1)experimental noise, 2)timing of sampling, 3) cell type, and 4)different filtering criteria are likely to explain some of the differences among these studies.

### Pathophysiology of the EPC response to SVCV infection

#### i. Oxidative stress in SVCV infection

Oxidative stress has been implicated in the pathogenesis of various neurodegenerative diseases, such as Alzheimer’s disease and Parkinson’s disease [19]. Oxidative stress occurs in cells when production of reactive oxygen species (ROS) exceeds the cell’s endogenous antioxidant defences. Many DNA and RNA viruses can trigger oxidative stress and induce host cell death in infected cells [20–22]. Upon SVCV infection, 19 genes involved in oxidative stress were regulated, including 11 down-regulated genes and 8 up-regulated genes (Table 3). The down-regulated genes included several subunits of the NADH dehydrogenase 1 alpha complex, the first enzyme complex in the electron transport chain located in the inner mitochondrial membrane, i.e. NADH dehydrogenase 1 alpha subcomplex 1(*NDUFA1*), *NDUFA7*, *NDUFA11* and *NDUFA12*, NADH dehydrogenase Fe-S protein 5 (*NDUFS5*), ubiquinol-cytochrome c reductase iron-sulphur subunit, and cytochrome b5 type A (*Cyb5a*). Cu/Zn-superoxide dismutase (*Cu/Zn SOD*), one of the major defences against ROS, was down-regulated more than 2 times post SVCV infection. The up-regulated genes included sestrin 3 (*sesn3*), uncoupling protein 2 (*UCP2*) and oxidative stress induced growth inhibitor 1 (*OSGIN1*). *Sesn3* encodes a member of the sestrin family of stress-induced proteins and could reduces the levels of intracellular reactive oxygen species [23]. Uncoupling protein 2 is a member of the larger family of mitochondrial anion carrier proteins and its main function is to control mitochondria-derived reactive oxygen species. *OSGIN1*, encoding an oxidative stress response protein, is regulated by p53 and is induced by DNA damage. *OSGIN1* also regulates apoptosis by inducing cytochrome c release from mitochondria. These regulated genes indicated that SVCV infection could induce oxidative stress in EPC cells resulting in a series of physiological changes. Another gene of interest is catalase, a common enzyme found in nearly all living organisms exposed to oxygen that is very important in protecting the cell from oxidative damage by ROS; but there was no detectable expression in EPC cells. Altogether, host and virus factors were both contributed to the induction of oxidative stress and the consequent cell damage.

**Table 3:** **List of the differentially expressed genes (DEGs) involved in the pathophysiology of the EPC response to SVCV infection**

Abbr.	Gene description	^*^Fold changes	^*^Fold changes	^*^Fold changes
		(3 h, Log2)	(6 h, Log2)	(24 h Log2)
**Oxidative stress in SVCV infection**				
NDUFA12	NADH dehydrogenase 1 alpha subcomplex 12	-	-	−1.36
NDUFA1	NADH dehydrogenase 1 alpha subcomplex 1	-	-	−1.76
NDUFA11	NADH dehydrogenase 1 alpha subcomplex subunit 11	−0.84	−1.01	−1.38
NDUFA7	NADH dehydrogenase 1 alpha subcomplex 7	-	-	−1.98
UQCRFS1	Ubiquinol-cytochrome c reductase iron-sulfur subunit	-	-	−2.51
ATPeVPL	Vacuolar ATP synthase 16 kDa proteolipid subunit	-	-	−1.22
NDUFS5	NADH dehydrogenase Fe-S protein 5	-	-	−1.11
COX7B	cytochrome c oxidase subunit VIIb	-	-	−1.25
SOD1	Cu/Zn-superoxide dismutase	-	-	−1.15
SESN	Sestrin-3	−1.61	−0.72	2.76
Cyb5a	Cyb5a protein	-	-	−3.06
ENC1	Ectodermal-neural cortex 1, a member of the kelch-related family of actin-binding proteins	1.30	-	−1.14
OSGIN1	Oxidative stress-induced growth inhibitor 1	-	-	1.39
UCP2	Mitochondrial uncoupling protein 2, control of mitochondria-derived reactive oxygen species	-	-	1.16
HSP90	Heat shock protein HSP 90-alpha	−1.88	−1.61	1.50
C/EBP beta	CCAAT/enhancer binding protein beta	0.98	-	2.96
GADD45A	Growth arrest and DNA-damage-inducible, alpha, a	−0.98	-	3.38
GADD45B	Growth arrest and DNA-damage-inducible, beta b	1.92	1.01	4.43
Jun-B	Transcription factor jun-B	1.73	2.14	4.43
**Apoptosis induced by SVCV infection**				
NFKBIA	NF-kappaB inhibitor alpha-like protein A	-	-	3.41
NFKBIB	NF-kappaB inhibitor alpha-like protein B	-	-	5.32
CSRNP1	Cysteine/serine-rich nuclear protein 1-like	1.17	0.69	3.99
CASP8	Caspase 8	-	-	1.35
TNFRSF1A	Tumor necrosis factor receptor superfamily member 1A precursor	-	-	1.09
BIRC2-3	Baculoviral IAP repeat-containing 3	0.90	-	1.43
MYD88	Myeloid differentiation primary response protein MyD88	1.45	-	2.02
GADD45A	Growth arrest and DNA-damage-inducible, alpha, a	−0.98	-	3.38
GADD45B	Growth arrest and DNA-damage-inducible, beta b	1.92	1.01	4.43
OSGIN1	Oxidative stress-induced growth inhibitor 1	-	-	1.39
YWHAE	Tyrosine 3-monooxygenase/tryptophan 5-monooxygenase activation protein, epsilon polypeptide 2 ,14-3-3 protein epsilon	-	-	−1.02
Gelsolin	Scinderin like b	-	-	−1.34
Mdm2	E3 ubiquitin-protein ligase	-	-	2.12
PDCD6	Programmed cell death protein 6	1.50	0.93	1.79
DDIT3	DNA damage-inducible transcript 3 protein	-	-	3.57
IRF7	IFN-regulatory factory 7	-	-	3.59
PIM1	Proto-oncogene serine/threonine-protein kinase pim-1	1.70	1.60	2.58
BBC3	BCL2 binding component 3	-	-	5.37
JUN-D	Transcription factor jun-D	-	-	5.45
Tax1bp1	Tax1-binding protein 1 homolog B	-	-	2.35
PHLDA3	Pleckstrin homology-like domain family B member 3-like	-	-	2.58
BIRC5	Baculoviral IAP repeat-containing protein 5	-	-	−2.13
PAWR	PRKC apoptosis WT1 regulator protein	-	-	−1.32
Mcl1b	Myeloid cell leukemia sequence 1	-	-	−1.13
Set	Protein SET	-	-	−1.03
**Regulation of the cytoskeleton by SVCV infection**				
ACTN1	Alpha-actinin-1	0.82	0.71	−1.43
ACTN4	Alpha-actinin-4	-	-	−1.45
ACTB	Beta-actin	-	-	−1.35
ARPC5	Actin related protein 2/3 complex, subunit 5A	-	-	−1.22
TUBA8	Tubulin, alpha 8 like 2	-	-	−3.32
TUBB6	Tubulin, beta 6 class V	-	-	−2.70
TUBA4	Tubulin alpha-4A chain-like	-	-	−3.08
TUBA1	Tubulin alpha-1C chain	-	-	−2.66
TUBA2	Tubulin, alpha 2	-	-	−2.19
TUBB	Tubulin beta-4 chain-like isoform 1	-	-	−2.04
TUBA	Tubulin alpha 6	-	-	−2.00
PFN21	PFN2l protein	-	-	−1.19
RRAS	Ras-related protein	-	-	−1.23
Gelsolin;	Scinderin like b	-	-	−1.34
RAC1	Ras-related C3 botulinum toxin substrate 1-like	-	-	−1.37
CFL	Cofilin 2, like	-	-	−1.17
PFN2	Profilin-2-like isoform 2	-	-	−1.37
GSN	GSNA	-	−0.78	−2.05
BRICK1	Probable protein BRICK1-like	-	-	−2.44
VCL	Vinculin	-	-	−1.34
FLNA;	Vilamin A	-	-	−1.73
COTL1	Voactosin-like protein	-	-	−2.21
ITGA11	Integrin alpha-11	0.73	1.55	2.01
**Inhibition of the interferon system**				
IFNR1	IFN-regulatory factor 1	-	-	4.77
IRF2	Interferon regulatory factor 2	-	-	2.23
IRFBP2	Interferon regulatory factor 2-binding protein 2-B	-	0.66	2.59
IRF7	IFN-regulatory factory 7	-	-	3.59

^*^Fold changes refers to the changes of gene expression in response to SVCV infection at the indicated time points, and the minus value means the gene is down-regulated after SVCV infection; while the positive value means the gene is up-regulated in SVCV-infected cells. The dashes (-) indicated that the expression level of certain gene is not changed at the indicated time point post SVCV infection when compared with 0 h post SVCV infection.

#### ii. Apoptosis induced by SVCV infection

Programmed cell death (apoptosis) is one of the most common forms of cell death in multicellular organisms and it plays a pivotal role during normal development and in the regulation of various physiological processes [24]. Two principle pathways of apoptosis exist in mammalian cells, i.e., the extrinsic or receptor-mediated pathway and the intrinsic pathway, which is mediated via mitochondrial and the endoplasmic reticulum. A previous study indicated that EPC cells infected with SVCV undergo apoptosis [6]. In agreement with those results, we found that 19 pro-apoptosis genes were up-regulated and 6 anti-apoptosis genes were down-regulated at 24 h post infection. Most of the up-regulated genes were involved in the death receptor pathway and the mitochondrial pathway, including *CASP8*, Bcl-2-binding component 3 (*BBC3*), tyrosine 3-monooxygenase/tryptophan 5-monooxygenase activation protein, epsilon polypeptide 2 (*YWHAE*), programmed cell death protein 6 (*PDCD6)* and many other genes involved in p53 signal transduction (*MDM2*, *GADD45* and *sestrin*). *CASP8* encodes an initiator caspase that directly cleaves downstream effector caspases such as caspase-3 or cleavaging Bid, a Bcl-2 family protein with a BH3 domain to initiate a mitochondrial amplification loop [25]. Upon SVCV infection, *CASP8* was up-regulated more than 2 times. *BBC3*, encoding an essential pro-apoptotic protein, was up-regulated more than 32 times at 24 h post infection. In the pathway of p53 dependent apoptosis, the up-regulation of *BBC3* can induce the expression of apoptosis regulator *BAX* and *BAK* to trigger apoptosis through the mitochondrial pathway. *MDM2*, an important negative regulator of the p53 tumour suppressor, was up-regulated more than 4 times upon SVCV infection. Meanwhile, several tumor necrosis factor (TNF) signal-related genes were up-regulated including tumour necrosis factor, tumour necrosis factor receptor superfamily member 1A/11A and 11B, and tumour necrosis factor alpha-induced protein 3/6. This indicated that the TNF signal was activated upon SVCV infection. Several genes involved in the p53 signal pathway were also regulated post SVCV infection. Taken together, SVCV infection could induce apoptosis through the TNF mediated extrinsic pathway and the mitochondrial pathway. Further studies are needed to reveal the detailed mechanism.

#### iii. Regulation of the cytoskeleton by SVCV infection

Viruses use different elements of the cytoskeleton for entry, replication, intracellular transport and budding [26]. Many studies have implicated microfilament, intermediate filaments, and microtubules, as well as proteins that regulate cytoskeleton functions, in the infectious cycles of viruses [27, 28]. Previous proteomic data revealed that SVCV infection could regulate the cytoskeleton of EPC cells. In our results, approximately 24 cytoskeleton- associated genes were down-regulated, including five microfilament-associated gene (*ACTA1*, *ACTA4*, *ACTB*, *FLNA*, and *ARPC5*) and several microtubule-associated genes *(TUBA2*, *TUBA4*, *TUBA8*, *TUB4*, and *TUB6*). These microtubule-associated genes were down-regulated more than 4 times. These genes are also associated with the functional category of cell communication. Among all the regulated genes, only one cytoskeleton-related gene (integrin, alpha 11, *ITGA11*) was up-regulated at 24 h post-viral infection. In agreement with the gene expression profile, EPC cells changed to a round sharp and lost the capacity to adhere at 24 h post-infection. Previous studies have shown that influenza A virus may interact with tubulin and induce disruption of the microtubule network and apoptosis in A549 cells [29]. We speculated that SVCV can also induce cytoskeletal disruption, which may be related to the release of SVCV particles, as well as induce apoptosis of infected cell, but this requires further study.

#### iv. Inhibition of the interferon system

The interferon (IFN) response is one of the most fundamental defence mechanisms against viral infection [30]. Viruses, which require cellular machinery for their replication, have evolved different strategies to counteract the action of IFN, particularly by the alteration of IFN-signaling and IFN-induced mediators that are required for virulence [31]. Here, four IFN-signaling genes were up-regulated and no IFN-stimulated genes (ISGs) were regulated untill 24 h post infection with SVCV. However, we could find many ISGs from the transcriptome of EPC cells, including MX dynamin-like GTPase 1 (*MX1*). These up-regulated genes include interferon regulatory factor 1 (*IRF1*), interferon regulatory factor 2 (*IRF2*), interferon regulatory factor 7 (*IRF7*), and IFR2 binding protein 2-B (*IBP2B*). The products of *IRF1* and *IRF2*, interferon regulatory factor 1 (IRF-1) and interferon regulatory factor 2 (IRF-2) are structurally similar but functionally distinct transcription factors that bind to the positive regulatory domains I and III (PRDI/III) within the human IFN-beta promoter [32]. IRF-1 serves as an activator of interferon alpha and beta transcription, while IRF-2 competitively inhibits the IRF-1-mediated transcriptional activation of interferon alpha and beta. Both of *IRF1* and *IRF2* were also up-regulated more than 4 times. More interestingly, IFR2 binding protein 2-B, which can interact with the C-terminal transcriptional repression domain of IRF-2, was up-regulated more than 4 times. The product of *IRF7*, interferon regulatory factor 7 (IRF-7), has been shown to play a role in the transcriptional activation of virus-inducible cellular genes, including the type I interferon genes [33]. *IRF7* was also up-regulated significantly at 24 h post SVCV infection. In summary, we speculate that SVCV infection might induce the production of IFN but block the function of IFN by inhibiting the production of ISGs through an unknown mechanism.

## Conclusions

In this study, we present the data from sequencing and assembly of the transcriptome of EPC cells at progressive times after SVCV infection. A great number of genes that were differentially expressed upon SVCV infection were obtained and functionally annotated. Further, the gene expression patterns for some of these genes were verified by RT-qPCR. The data present here demonstrate previously unrecognised changes in gene transcription that are associated with SVCV infection in vitro, and many potential cascades identified in the study clearly warrant further experimental investigation. Our data also provide new clues to the mechanism of viral susceptibility in EPC cells.

## Methods

### Cells and virus stock

The EPC cell line (ATCC: CRL-2872) was maintained in Eagle’s minimum essential medium (MEM, Invitrogen, Carlsbad, CA) supplemented with 10% foetal calf serum at 25°C. SVCV (ATCC: VR -1390), as originally isolated by Fijan et al. [34], was kindly provided by Professor Yuanan Lu (Department of Public Health Sciences, John A. Burns School of Medicine, University of Hawaii at Manoa). Virus multiplication and titration assays were performed as described previously [6].

### SVCV infection and sample collection

For viral infection assays, the SVCV stock was diluted and then used to infect EPC cells at a multiplicity of infection (MOI) of 1.0. Following 1 h of viral adsorption at 25°C, the inoculum was removed, cells were washed twice with PBS (pH7.2), and normal medium (MEM containing 10% FBS) was added. Cells were cultivated at 25°C for the time points (0 h, 3h, 6 h, and 24 h) indicated in the figure legends, and then further processed. At each time point in the viral infection assay, three parallel samples were prepared as biological replicates.

### RNA extraction, mRNA purification, and cDNA synthesis

Total RNA was extracted from EPC cells at 0, 3, 6, and 24 h postinfection in three independent experiments, using TRIzol reagent (Invitrogen, Carlsbad, CA) according to the manufacturer’s protocol. The concentration of total RNA was determined using NanoDrop (Thermo Scientific, Waltham, MA), and the RNA integrity value (RIN) was checked using the RNA 6000 Pico LabChip on an Agilent 2100 Bioanalyzer (Agilent, Santa Clara, CA). For mRNA purification, total RNA was incubated with 10 U DNase I (Ambion, Grand Island, NY) at 37°C for 1 h, followed by a purification step using a MicroPoly (A) Purist Kit (Ambion, Grand Island, NY) according to the manufacturer’s instructions. Then, the purified mRNA was dissolved in RNA storage solution, and the final concentration was determined using NanoDrop. Double-stranded cDNA was synthesised from mRNA according to Ng’s full-length cDNA synthesis protocol with some modifications [35]. A GsuI-oligo dT primer was used for the first-strand cDNA synthesis with 10 mg of mRNA and Superscript II reverse transcriptase (Invitrogen, Carlsbad, CA). After incubation at 42°C for 1 h, the 5’- CAP structure of mRNA was oxidised by NaIO4 (Sigma, St. Louis, MO) and ligated to biotin hydrazide, which was used to select complete mRNA/cDNA heterodimers by binding to Dynal M280 beads (Invitrogen, Carlsbad, CA). After the second strand cDNA synthesis, the polyA and 5’- adaptor were removed by GsuI digestion.

### cDNA sequencing

The cDNA was sonicated to the range of 300-500 bp and purified using Ampure beads (Agencourt, USA). The cDNA libraries were prepared with a TruSeq™ DNA sample Prep Kit – Set A and were PCR-amplified (15cycles) using a TruSeq PE Cluster kit (Illumina); there were then sequenced on a Genome Analyzer II (Illumina) according to the manufacturer’s instructions.

### Sequence assembly and annotation

Raw reads were first cleaned by removing adaptor sequences and low quality sequences (Q > 20), and then assembled into EST clusters (contigs) using Trinity with the default assembly parameters (http://trinityrnaseq.sourceforge.net/). The raw data from the Illumina reads have been deposited into NCBI’ s Sequence Read Archive (SRA) under the accession numbers of SRX546183, SRX546212, SRX546380, SRX547280, and SRX547883.

All contigs were annotated with GetORF from the EMBOSS package (http://emboss.sourceforge.net/) [36]. The ORF of each predicted protein was used for BLASTp searches, against the Swiss-Prot and the NCBI nr databases, setting the e-value threshold to 10^−5^. GO annotations were also derived based on sequence similarity with GoPipe (http://www.geneontology.org/) [37]. The COG and KEGG pathway annotations were performed using Blastall software against the Cluster of Orthologous Groups database and the Kyoto Encyclopaedia of Genes and Genomes database (http://www.genome.jp/kegg/) [38]. In this study, we used the default parameters in each approach and no other custom approach was used.

### Analysis of gene expression profiles by digital gene expression tag profiling

To analyse genes that were differentially expressed at different stages of infection, the number of reads for each of the contigs from the indicated samples was converted to Reads per Kilobase per Million (RPKM) [39]. Then, the MA-plot-based method with Random Sampling (MARS) model in the DEGseq package (http://www.bioconductor.org/packages/release/bioc/html/DEGseq.html) was used to calculate the expression abundance of each contig among the indicated samples [40]. We used an false discovery rate (FDR) to determine the threshold of p value for this analysis. An FDR of 0.001 was considered to have significant expression abundance. For the identification of the pathways that the differentially expressed genes (DEGs) are predicted to participate in, all DEGs were mapped to terms in the KEGG database and searched for significantly enriched KEGG terms compared to the genomic background.

### Analysis of gene expression by real-time PCR

Eight genes were selected for the confirmation of DEG data by real-time PCR, using the SYBR Premix Ex Taq kit (Takara, Japan) according to the manufacturer’s instructions in a StepOne machine (Applied Biosystems, Carlsbad, CA). Quantification was performed using the comparative C_T_ method with separate reaction tubes for chosen DEGs and reference (TATA box binding protein, *TBP*) RNAs. Primers for qPCR were designed with Primer Express software (version 3.0, ABI) based on the target sequences. The primers used for qPCR of the selected DEGs are listed in Additional file 2: Table S1. All reactions were performed in a 10 μl volume (5 μl, 2 × SuperMix Universal; 200 nM of each forward and reverse primer; and 0.2 μl ROX reference Dye). A total of 40 cycles were performed. All samples were analysed in triplicate and the relative gene expression data were expressed as the transcription units relative to those of the *TBP* gene using the 2^-CT^ method [41]. Three parallel experiments were performed and one representative experiment of three is shown.

## Electronic supplementary material

Additional file 1: Table S2.: List of the differentially expressed genes (DEGs) in EPC cells between SVCV infection at 24 h and 0 h. (XLS 124 KB)

Additional file 2: Table S1.: Primers used for RT-qPCR verification of DEG data. **Table S3**. List of the homologous genes involved in viral susceptibility that lacked detectable expression levels in the EPC transcriptome. **Table S4**. List of the differentially expressed genes in SVCV-infected EPC cells which were both identified by MALDI-TOF/TOF and mRNA-sequencing. (DOC 113 KB)

## Abbreviations

SVCV: Spring viraemia of carp virus
SVC: Spring viraemia of carp
EPC: Epithelioma papulosum cyprinid
GO: Gene ontology
KOG: EuKaryotic Orthologous Groups
KEGG: Kyoto Encyclopedia of Genes and Genomes
DEGs: Differentially expressed genes
TBP: TATA box binding protein.
