# Supplementary material for: Transcriptome analysis of epithelioma papulosum cyprini cells after SVCV infection
Source: BMC Genomics. 2014 Oct 25;15(1):935. doi: 10.1186/1471-2164-15-935 (PMC4221675; doi:10.1186/1471-2164-15-935)
Supplement: Supplementary file 2 — Additional file 2: Table S1.: Primers used for RT-qPCR verification of DEG data. Table S3. List of the homologous genes involved in viral susceptibility that lacked detectable expression levels in the EPC transcriptome. Table S4. List of the differentially expressed genes in SVCV-infected EPC cells which were both identified by MALDI-TOF/TOF and mRNA-sequencing. (DOC 113 KB) [file 12864_2014_6624_MOESM2_ESM.doc]

**Additional file 1**

**Table S1. Primers used for qRT-PCR verification of DEG data.**

| Target genes | | Primers (5’-3’) |
| --- | --- | --- |
| *c-fos* | Proto-oncogene c-Fos | ACGCTGCAATCTGAAACTGA |
|  |  | GGCGAGGATGAACTCTAACC |
| *c-Jun* | Transcription factor AP-1 | ATGGAGAGTCAGGAGCGGAT |
|  |  | GTTCTGCGACTTTAGGTTCTTG |
| *CASP8* | Caspase 8 | ATGGTGAACCGGTTGAAATCC |
|  |  | CCTGACAGGCCTGAATGAAGA |
| *MYD88* | Myeloid differentiation primary response protein | GTAAGAGGATGGTGGTGGTA |
|  |  | GTAGACAACAGGGATTAGGC |
| *KLF2A* | Kruppel-like factor 2α | GTTGCGGCTGGAAGTTTG |
|  |  | ATGGAGGGCGAGATGGTC |
| *UQCRFS1* | Cytochrome b-c1 complex subunit Rieske, mitochondrial | ACCATAGTCACCAGCATTAG |
|  |  | GACAAAGACCGTGTTCAGA |
| *HSP47* | Heat shock protein 47 | CGGTCTCTGTTCCAATGATG |
|  |  | CATGCTGGACTTCTTATGTGC |
| *GSNA* | GSNA | GAAGTTTGATTTGGTGGCAG |
|  |  | AGTGGAGGTCGTACTGTAGGTT |
| *TBP* | TATA box binding protein | TTACCCACCAGCAGTTTAG |
|  |  | ACCTTGGCACCTGTGAGTA |

**Table S3. List of the homologous genes involved in viral susceptibility that lacked detectable expression levels in the EPC transcriptome**.

| Gene ID | Symbol | Gene Description |
| --- | --- | --- |
| 84675 | TRIM55 | Tripartite motif-containing 55 |
| 29882 | ANAPC2 | anaphase promoting complex subunit 2 |
| 5583 | PRKCH | protein kinase C,eta |
| 4501 | MT1X | metallothionein 1X |
| 91074 | ANKRD30A | ankyrin repeat domain 30A |
| 727851 | RGPD8 | RANBP2-like and GRIP domain containing 8 |
| 3431 | SP110 | SP110 nuclear body protein |
| 3020 | H3F3A | H3 histone, family 3A |
| 1950 | EGF | epidermal growth factor (beta-urogastrone) |
| 57626 | KLHL1 | Kelch-like 1 (Drosophila) |
| 140730 | RIMS4 | regulating synaptic membrane exocytosis 4 |
| 4277 | MICB | MHC class Ⅰpolypeptide-related sequence B |
| 5046 | PCSK6 | proprotein convertase subtilisin/kexin type 6 |
| 3552 | IL1A | Interleukin 1,alpha |
| 54205 | CYCS | Cytochrome c,somatic |
| 3111 | HLA-DOA | major histocompatibility complex,class Ⅱ,DO alpha |
| 6382 | SDC1 | syndecan 1 |
| 8600 | TNFSF11 | tumor necrosis factor (ligand) superfamily,member 11 |
| 57576 | KIF17 | Kinesin family member 17 |
| 81494 | CFHR5 | complement factor H-related 5 |
| 10537 | UBD | ubiquitin D |
| 5413 | SEPT5 | septin 5 |
| 121504 | HIST4H4 | histone cluster 4,H 4 |
| 8740 | TNFSF14 | tumor necrosis factor (ligand) superfamily,member 14 |
| 3122 | HLA-DRA | major histocompatibility complex,class Ⅱ,DR alpha |
| 348 | APOE | apolipoprotein E |
| 3105 | HLA-A | major histocompatibility complex,class Ⅰ,A |
| 929 | CD14 | CD14 molecule |
| 914 | CD2 | CD2 molecule |
| 965 | CD58 | CD58 molecule |
| 847 | CAT | catalase |
| 3383 | ICAM1 | Intercellular adhesion molecule 1 |
| 6693 | SPN | sialophorin |
| 960 | CD44 | CD44 molecule(Indian blood group) |
| 942 | CD86 | CD86 molecule |
| 10890 | RAB10 | RAB10,member RAS oncogene family |
| 10134 | BCAP31 | B-cell receptor-associated protein 31 |
| 3609 | ILF3 | Interleukin enhancer binding factor 3.90kDa |
| 250 | ALPP | alkaline phosphatase,placental(Regan isozyme) |
| 7314 | UBB | ubiquitin B |
| 5757 | PTMA | prothymosin,alpha |
| 1510 | CTSE | cathepsin E |
| 3268 | AGFG2 | ArfGAP with FG repeats 2 |
| 4153 | MBL2 | mannose-binding lectin(protein C)2,soluble(opsonic defect) |
| 4246 | SCGB2A1 | secretoglobin, family 2A, member 1 |
| 7535 | ZAP70 | zeta-chain (TCR) associated protein kinase 70kDa |
| 64600 | PLA2G2F | phospholipase A2, group IIF |
| 283748 | PLA2G2D | phospholipase A2, group IVD(cytosolic) |
| 8534 | CHST1 | Carbohydrate (keratan sulfate Gal-6) sulfotransferase 1 |
| 4502 | MT2A | metallothionein 2A |
| 920 | CD4 | CD4 molecule |
| 7852 | CXCR4 | chemokine (C-X-C motif) receptor 4 |
| 3107 | HLA-C | major histocompatibility complex,class Ⅰ,C |
| 213 | ALB | albumin |
| 1267 | CNP | 2’,3’-cyclic nucleotide 3’ phosphodiesterase |
| 3178 | HNRNPA1 | heterogeneous nuclear ribonucleoprotein A1 |
| 5970 | RELA | v-rel reticuloendotheliosis viraloncogene homolog A (avian) |
| 1654 | DDX3X | DEAD (Asp-Glu-Ala-Asp) box polypeptide 3,X-linked |

**Table S4. List of the differentially expressed genes in SVCV-infected EPC cells which were both identified by** **MALDI-TOF/TOF and mRNA-sequencing.**

| Symbol | Gene Description | *Fold changes (Log2)identified by Sequencing | *Fold changes identified (Log2) MALDI-TOF/TOF |
| --- | --- | --- | --- |
| ACTB | actin, beta | -1.35^C^ | -1.77^a^ |
| ACTN1 | alpha-actinin-1 | -1.43^C^ | 3.01^a^ |
| CFL2L | cofilin 2, like | -1.17^C^ | 3.22^c^ |
| HSP4 | heat shock protein 4 | 1.01^C^ | -1.43^a^ |
| HSPA8 | heat shock cognate 71 kDa protein | -1.33^B^ | -1.45^a^ |
| HSP90 | heat shock protein 90 | -1.61^B^ | -1.40^a^ |
| PGD | 6-phosphogluconate dehydrogenase, decarboxylating | 0.43^A^ | -2.36^a^ |
| HMGCS | 3-hydroxy-3-methylglutaryl-coenzyme A synthase | -2.12^B^ | 3.91^a^ |
| DDB | protein phosphatase 2 | 5.78^C^ | -2.41^c^ |
| CNN2 | calponin-2 | -1.96^C^ | 1.55^b^ |

^*^Fold changes is the changes of gene expression in response to SVCV, and the minus value means the gene is down-regulated after SVCV infection; while the positive value means the gene is up-regulated in SVCV-infected cells.

A/B/C represents the time point of 3 h, 6 h, and 24 h post SVCV infection in this study. While a/b/c indicates that the differentially protein identified by MALDI-TOF/TOF post SVCV infection at 24 h, 48 h, and 72 h in Reference [10].
